# Stimulating myelin restoration with BDNF: a promising therapeutic approach for Alzheimer's disease

**DOI:** 10.3389/fncel.2024.1422130

**Published:** 2024-09-02

**Authors:** Ioanna Zota, Konstantina Chanoumidou, Achille Gravanis, Ioannis Charalampopoulos

**Affiliations:** ^1^Department of Pharmacology, Medical School, University of Crete, Heraklion, Greece; ^2^Institute of Molecular Biology and Biotechnology, Foundation of Research and Technology-Hellas (IMBB-FORTH), Heraklion, Greece

**Keywords:** Alzheimer's disease, myelin degeneration, oligodendrocytes, neurotrophins, BDNF

## Abstract

Alzheimer's Disease (AD) is a chronic neurodegenerative disorder constituting the most common form of dementia (60%−70% of cases). Although AD presents majorly a neurodegenerative pathology, recent clinical evidence highlights myelin impairment as a key factor in disease pathogenesis. The lack of preventive or restorative treatment is emphasizing the need to develop novel therapeutic approaches targeting to the causes of the disease. Recent studies in animals and patients have highlighted the loss of myelination of the neuronal axons as an extremely aggravating factor in AD, in addition to the formation of amyloid plaques and neurofibrillary tangles that are to date the main pathological hallmarks of the disease. Myelin breakdown represents an early stage event in AD. However, it is still unclear whether myelin loss is attributed only to exogenous factors like inflammatory processes of the tissue or to impaired oligodendrogenesis as well. Neurotrophic factors are well established protective molecules under many pathological conditions of the neural tissue, contributing also to proper myelination. Due to their inability to be used as drugs, many research efforts are focused on substituting neurotrophic activity with small molecules. Our research team has recently developed novel micromolecular synthetic neurotrophin mimetics (MNTs), selectively acting on neurotrophin receptors, and thus offering a unique opportunity for innovative therapies against neurodegenerative diseases. These small sized, lipophilic molecules address the underlying biological effect of these diseases (neuroprotective action), but also they exert significant neurogenic actions inducing neuronal replacement of the disease areas. One of the significant neurotrophin molecules in the Central Nervous System is Brain-Derived-Neurotrophin-Factor (BDNF). BDNF is a neurotrophin that not only supports neuroprotection and adult neurogenesis, but also mediates pro-myelinating effects in the CNS. BDNF binds with high-affinity on the TrkB neurotrophin receptor and enhances myelination by increasing the density of oligodendrocyte progenitor cells (OPCs) and playing an important role in CNS myelination. Conclusively, in the present review, we discuss the myelin pathophysiology in Alzheimer's Diseases, as well as the role of neurotrophins, and specifically BDNF, in myelin maintenance and restoration, revealing its valuable therapeutic potential against AD.

## Highlights

- Myelin degeneration impact in Alzheimer's disease progress.
- Targeting of the BDNF/TrkB receptor to promote oligodendrogenesis and remyelination.
- BNDF-mimetics as a novel therapeutic approach for Alzheimer's disease.

## 1 Introduction

Alzheimer's Disease (AD) is a chronic devastating neurodegenerative disorder, constituting approximately 70% of dementia cases and standing as a major global public health priority, as acknowledged by the World Health Organization (Prince et al., 2015; Lane et al., 2018). Over the age of 65, 1% of the world's population is affected (Alzheimer's Association, 2024). AD is seen as a multifaceted disease with a complicated pathophysiology that is brought on by a number of risk factors, including genetic background and environmental factors (Armstrong, 2019). Aging is the main risk factor for the progression of AD (Hou et al., 2019). From a clinical point of view the cognitive decline observed at a later phase in AD patients is the main feature of the disease (Pramanik et al., 2017). The defining histological features of the disease until now are the accumulation of toxic amyloid-beta aggregates and the formation of neurofibrillary tangles (NFTs) of hyperphosphorylated tau protein (Reiss et al., 2018; Gao et al., 2022; Breijyeh and Karaman, 2020). Other hallmarks include neuropil threads, dystrophic neurites, associated astrogliosis, and microglial activation (Serrano-Pozo et al., 2011; Lane et al., 2018). The downstream effects of these pathological processes are synaptic loss and massive neuronal death, resulting in neurodegeneration and brain atrophy (Schneider et al., 2009). To date, there are only symptomatic therapies with limited efficacy, while there is no effective cure.

Recent studies highlight myelin breakdown as a critical early event in AD which exacerbating its progression. Single-cell transcriptomic analyses have detected gene expression changes in oligodendrocyte lineage cells from early-stage AD patient brains related to negative regulation of oligodendrocyte differentiation and myelination (Mathys et al., 2019; Zhou et al., 2021). Myelin abnormalities have been identified in the brain of AD animal models, including triple-transgenic AD (3xTg-AD) and APP/PS1 mice (Desai et al., 2009, 2010; Mitew et al., 2010; Schmued et al., 2013). Recently, a causative correlation between demyelination, Amyloid-β deposition and microglia disturbance has been identified in a single-cell manner in human AD brains and 5xFAD mice. Specifically, single-cell RNA sequencing revealed that myelin damage exacerbates Aβ plaque formation, which in turn leads to microglial activation and neuroinflammation. This mechanistic insight provides a deeper understanding of how myelin dysfunction contributes to the pathogenesis of AD and highlights potential therapeutic targets for early intervention (Depp et al., 2023). However, it remains unclear whether myelin loss is due to higher susceptibility of, reduced myelin repair capacity, or exposure to external toxic stimuli. Thus, several pathological aspects of AD, such as APP/Aβ pathways, APOE4 and lipid metabolism, tau-dependent pathologies and even PI3K signaling or iron metabolism are under investigation as potential therapeutic targets due to their effects in myelin homeostasis (Hardy and Selkoe, 2002; Mahley and Huang, 2012; Ballatore et al., 2007; Talbot and Wang, 2014; Smith et al., 2010).

Neurotrophins constitute a family of secreted polypeptides encompassing NGF, BDNF, NT-3 and NT-4 that are well known for their neuroprotective properties. They regulate neuronal growth, survival and synaptogenesis in the adult brain via binding to specific receptors including Trks and p75NTR. Apart for their neuronal-related functions neurotrophins are critical for the maintenance of brain homeostasis by regulating other brain cell types including oligodendrocytes. More specifically, brain-derived neurotrophic factor (BDNF) is known to selectively act through the TrkB receptor, promoting axon remyelination and OPC proliferation, and differentiation. Furthermore, BDNF enhances myelination in the CNS and tends to increase the density of OPCs both *in vitro* and *in vivo* (McTigue et al., 1998; Du et al., 2003; Fulmer et al., 2014; Wong B. X. et al., 2014). Recent studies show that BDNF is crucial for neurogenesis and neuronal plasticity by promoting the proliferation and differentiation of neural progenitor cells, enhancing synaptic strength, and modulating dendritic spine structure, which are vital for cognitive functions and recovery from neurodegenerative diseases. Additionally, BDNF levels are positively influenced by physical exercise and antidepressant treatments, further supporting its role in mental health and brain resilience.

αNumerous studies have demonstrated that the processing and expression levels of neurotrophins are dysregulated in AD, contributing significantly to the pathology of the condition. In the present review we discuss the complex interplay between BDNF signaling impairment, myelin disruption, and the progression of Alzheimer's disease, as well as the therapeutic potential of BDNF targeting in establishing a re-myelination approach in AD.

## 2 Myelin impairment in AD

Recent studies characterize myelin breakdown as an aggravating factor in AD, in addition to the formation of amyloid plaques and neurofibrillary tangles that are to date the main pathological hallmarks of the disease. Oligodendrocytes (OL) are the myelinating cells of the CNS ensuring the rapid transmission of signals between nerve cells, while supporting their metabolic needs, and assuring a major neuroprotective role. Notably, significant reduction has been observed in the number of oligodendrocytes in the black and white matter (WM) of AD patients (Sun et al., 2017). Additionally, myelin loss and oligodendrocyte lesions appear as an early symptom of AD (Nasrabady et al., 2018). Histopathological analyses of AD patients reveal demyelination and axonal damage, potentially causing functional disconnections between brain regions along with specific gray matter structural defects (Delbeuck et al., 2003). Although WM studies in preclinical AD are limited, they present various findings, including regional increases and decreases in indices of WM integrity, proving early alterations in WM in AD patients (Chao et al., 2013; Racine et al., 2014).

In AD, myelin loss is a common finding, contributing to cognitive impairment (Behrendt et al., 2013; Zhan et al., 2015). Research indicates focal demyelination and oligodendrocyte loss localized to Aβ plaques in AD patients (Mitew et al., 2010). Significant white matter loss, particularly in later-myelinating regions like the temporal and frontal lobes, is associated with AD pathology (Stricker et al., 2009). Myelin breakdown in these regions may release iron, promoting Aβ pathology, which exacerbating myelin loss (Bartzokis et al., 2007). This process contributes to the formation of toxic Aβ fibrils residing in the brain and fostering amyloid plaque development (Bartzokis et al., 2007). Oligodendrocyte dysfunction induced by toxic Aβ could also have a potential aggravating role in neuronal apoptosis observed during the AD progression besides other signals that are involved in neurodegeneration (Lee G. et al., 2004; Alberghina and Colangelo, 2006). In turn, Aβ has been found to induce myelin injury, particularly in regions with late myelin damage, inhibiting remyelination by adult OPCs (Horiuchi et al., 2012). Focal myelin injury is observed in the core of Aβ plaques, and age-related demyelination correlates with cognitive impairment in AD (Mitew et al., 2010; Kavroulakis et al., 2018). Myelin changes, especially in the frontal cortex, emerge as early pathological feature that correlates to cognitive decline (Grydeland et al., 2013). Despite these insights, the relationship between Aβ pathology and myelin alterations remains incompletely understood, necessitating further research for a more comprehensive understanding.

In 5xFAD transgenic mice, an experimental model of Alzheimer's disease, demyelination has been observed to precede the accumulation of amyloid plaques (Wu et al., 2018) and increases over time, especially in areas associated with cognitive activity (Gu et al., 2018). On the contrary although Depp and colleagues showed that myelin deficits induce amyloid-β deposition they did not report any significant decrease in overall myelination levels in the gray and white matter among wild-type, 5xFAD, and APPNLGF mice at 6 months of age despite the high density of amyloid plaques at this stage (Depp et al., 2023). Alterations in OPC and oligodendrocyte numbers have been observed in various models of Alzheimer's disease, although findings vary, likely due to differences in transgenic animal models, ages of analysis and analyzed markers. A decrease in the number of NG2+ OPCs has been noted in the hippocampus of 9-month-old APP/PS1 mice (Chacon-De-La-Rocha et al., 2020), mirroring findings of reduced OPCs in the cortex of postmortem AD specimens (Behrendt et al., 2013). Conversely, an increase in OLIG2+ OPCs has been reported in 6–8-month-old APP/PS1 mice, followed by a reversal of myelin loss at 9 months (Behrendt et al., 2013). However, recent analysis of 12-month-old APP/PS1 animals revealed unaltered oligodendrogenesis but significant OL loss in the hippocampus (DeFlitch et al., 2022). Similarly, a study of 3xTag-AD mice indicated a reduced number of myelinating OLs with unchanged numbers of immature OLs (Desai et al., 2010). Notably, a recent study by Dr. Nave's lab demonstrated the causal effects of demyelination on amyloid-β aggregation, inflammatory exacerbation, and AD progression (Depp et al., 2023). Likewise, previous study of our group reported demyelination and impairment of OPC populations in the hippocampus of 6-month-old 5xFAD mice (Zota et al., 2024). Therefore, better understanding of oligodendrogenesis dynamics is imperative to pinpoint the appropriate time window for potential therapeutic interventions targeting the control of demyelination and re-myelination in AD.

The inability of oligodendrocyte precursors (OPCs) to remyelinate neuronal axons is probably caused by the presence of a plethora of inhibitory factors that target the myelination process (Lourenço et al., 2016). Oligodendrocytes and oligodendrocyte progenitor cells (OPC) are vulnerable to multiple pathological conditions met in AD brain, including β-amyloid, inflammation, and oxidative stress (Pang et al., 2003; French et al., 2009; Zhang et al., 2019). Whether myelin loss is solely a secondary effect to OL death or is also attributed to abnormalities in oligodendrogenesis and/or remyelination capacity remains poorly understood.

## 3 Myelin regeneration: a potential novel target in AD therapy

In addition to facilitating saltatory conduction via myelination, oligodendrocytes provide metabolic support to neurons. Oligodendrocytes provide lactate and pyruvate to axons, essential substrates for ATP production during periods of high neuronal activity (Fünfschilling et al., 2012). They also maintain axonal integrity by modulating redox balance and offering protection against oxidative stress (Saab et al., 2021). Furthermore, oligodendrocytes facilitate the removal of extracellular potassium ions and neurotransmitters, maintaining homeostasis in the neural environment (Zhang, 2023). This metabolic regulatory role is crucial for sustaining long-term neuronal function and protect them from neurodegenerative insults, underscoring the dynamic and vital role of oligodendrocytes in CNS homeostasis (Philips and Rothstein, 2017).

Neuronal myelination is vital for enhancing the intricate cognitive functions of the central nervous system and facilitating sophisticated network integration within the brain. Myelin breakdown results in neuronal dysfunction and a decline in cognitive abilities. Inhibition of Lingo-1, a receptor that negatively regulates remyelination, can attenuate memory deficits in the 5xFAD mouse model of AD, suggesting its potential utility in managing the progression of the disease (Wu et al., 2018). Postmortem examinations of AD brains and genetic studies have linked myelin impairments to Alzheimer's disease. Growing evidence connect myelin impairment with the presence of amyloid-beta plaques and tau hyperphosphorylation (Mitew et al., 2010; Zhan et al., 2015; Rubinski et al., 2024). Additionally, the apolipoprotein E4 allele (ApoE4) may play roles in myelin impairments seen in AD patients primarily affecting cholesterol-rich myelin sheath formation (Cheng et al., 2022). Furthermore, ApoE directly enhances the maturation of OPCs and oligodendrocytes, improving AD-related cognitive function (Santos-Gil et al., 2021). Decreased neuronal activity, elevated Aβ levels, and inflammation are all factors contributing to myelin damage in AD patients. The formation of amyloid plaques from fractions of damaged/altered cells exaggerates those symptoms, further damaging both neurons and glial cells. These events combined with remyelination deficiency ultimately lead to the cognitive decline and brain deterioration in AD (Tse and Herrup, 2017). Importantly, therapies aimed at promoting remyelination have shown promise in restoring neuronal function and enhancing cognition (Geraghty et al., 2019; Zhang et al., 2019). For example, remyelination impairment and associated cognitive deficits can be rescued by the intervention of a small-molecule TrkB agonist acting upon oligodendrocyte progenitor cells (OPCs) (Geraghty et al., 2019). Consequently, novel strategies that promote OPC differentiation and myelin regeneration present a potential complementary therapeutic approach for AD patients.

## 4 Candidate targets to enhance remyelination in AD

Oligodendrocyte progenitor cells constitute approximately 5% of the adult brain. Adult OPCs retain their proliferative and differentiation capacity into mature oligodendrocytes throughout adulthood although to a lesser extent (Beiter et al., 2022). *In vivo* and *in vitro* studies have revealed numerous factors regulating OPCs differentiation, oligodendrocytes maturation and myelination. Among these factors LINGO-1 and hyaluronan act as negative regulators and BDNF as positive regulator of these processes (Emery, 2010; Snaidero and Simons, 2014, 2017; Emery and Lu, 2015; Takebayashi and Ikenaka, 2015; Bergles and Richardson, 2016; Mayoral and Chan, 2016; Wheeler and Fuss, 2016). Additionally, many molecular factors linked to AD pathophysiology have been correlated with impaired myelination and represent attractive targets for intervention.

### 4.1 Aβ oligomers and amyloid precursor protein

Oligodendrocyte-lineage cells, similar to other cell types in the brain, are notably rich in amyloid precursor protein (APP) expression, underscoring the importance of APP beyond neuronal functions. Despite being relatively understudied, the roles of APP in non-neuronal cells, including oligodendrocytes, are gaining recognition. These cells express both non-amyloidogenic and amyloidogenic enzymes at levels comparable to neurons, suggesting that APP processing pathways in oligodendrocytes may have significant implications for brain health and disease (Haass and Selkoe, 2023; Johnson and Chiu, 2023). For instance, the non-amyloidogenic pathway, which predominates under physiological conditions, generates soluble APPα (sAPPα), a neuroprotective fragment that promotes neurite outgrowth and synaptic plasticity. Conversely, the amyloidogenic pathway can lead to the production of Aβ peptides, which are implicated in the pathogenesis of Alzheimer's disease. This highlights the need for further research to elucidate the specific functions and regulatory mechanisms of APP in oligodendrocyte-lineage cells.

Recent studies indicate that APP and its metabolites may play crucial roles in oligodendrocyte development and myelination. APP expression in oligodendrocytes has been associated with the regulation of cell proliferation, differentiation, and survival. Moreover, oligodendrocyte-derived APP and its cleavage products might interact with neuronal APP, influencing axonal integrity and function (Johnson and Chiu, 2023). This interaction highlights a complex cross-talk between neurons and oligodendrocytes, mediated by APP signaling pathways, which could be pivotal in maintaining CNS homeostasis and in the context of neurodegenerative diseases.

Soluble Aβ oligomers in Alzheimer's disease exhibit dual effects on myelination. On the one hand, Aβ inhibits oligodendrocyte survival and impedes myelin sheath formation but on the other hand it can induce myelin basic protein expression, promoting oligodendrocyte differentiation and maturation (Horiuchi et al., 2012; Quintela-López et al., 2019). Although reducing Aβ toxicity rescues myelin integrity, regeneration remains unaffected. Compounds like low-sulfated modified heparin mimetics can bind to Aβ, preventing its inhibition of oligodendrocyte precursor cell differentiation and facilitating remyelination (Fleming et al., 2007; McCanney et al., 2019). The amyloid precursor protein (APP) is implicated upstream of Aβ plaque deposition in this context. Knocking out APP results in significantly delayed or no remyelination (Truong et al., 2019). In a transgenic mouse model with amyloid pathology, early Aβ plaque deposition stages are associated with increased oligodendrocyte precursor cells (OPCs) and their subsequent differentiation into mature oligodendrocytes (OLs) (Behrendt et al., 2013). However, excessive APP may impair remyelination, as evidenced by decreased OLs in human AD postmortem tissues. The myelin repair mechanism may involve Arginase 1 (Arg1) expression, as demonstrated by RNA transcriptome analysis and cell type-profiling in APP mice, revealing a significant association between insufficient Arg1 expression in myelin-producing oligodendrocytes and subsequent neurodegeneration and Aβ deposition (Ma et al., 2021). Interestingly, Arg1 deficiency promotes OLs migration and upregulates genes related to myelination process but upregulates pro-inflammatory markers, and its reduced levels in demyelination further implicate Arg1 deficiency in overall myelin pathology (Bruce et al., 2018). Furthermore, the overexpression of Arg1 in the CNS is capable of reducing the inflammatory response and improving tau pathology (Hunt et al., 2015). Another study found that Arg1-positive microglia decreased Aβ plaque burden in an IL-1β-dependent inflammatory environment (Cherry et al., 2015). Additionally, a myeloid-specific knockout of Arg1 in a mouse model of retinal injury led to increased neuronal loss and heightened inflammatory responses (Fouda et al., 2018). The aforementioned findings suggest a crucial role for proper Arg1 function in both normal conditions and pathological challenges associated with amyloidosis. Hence, understanding the pathways that regulate Arg1 metabolism may offer new therapeutic opportunities to rebalance immune function and enhance the health of microglia and macrophages.

APP undergoes enzymatic processing through downstream pathways, involving α-secretases like ADAM10 and ADAM17, resulting in the formation of the non-neurotoxic soluble APP alpha (sAPPα) (Seals and Courtneidge, 2003). Elevated sAPPα, produced by α-secretase, exhibits reparative and protective effects in demyelination. In a demyelination mouse model, the administration of etazolate, a sAPPα promoter, restored damaged myelin, increased myelin basic protein (MBP) and mature oligodendrocytes (OLs), and provided protection against further demyelination (Llufriu-Dabén et al., 2018). The FDA-approved acetylcholinesterase inhibitor (AChEI) rivastigmine, known also to enhance α-secretase processing, has demonstrated efficacy in promoting sAPPα production in both 3 × TG mice and human post-mortem tissues (Ray et al., 2020). However, there is evidence suggesting that rivastigmine does not directly impact oligodendrogenesis (Cui et al., 2019). Amyloid precursor protein (APP) can undergo cleavage by β-secretase, specifically BACE-1, resulting in the formation of amyloid-beta (Aβ) (Vassar et al., 1999). BACE-1 is also responsible for cleaving neuregulin 1 (NRG1) protein that is essential for initiating remyelination. Aged APP/PSEN1 transgenic mice with vascular pathology and their non-APP aged stroked counterparts exhibit chronic upregulation of BACE1/NRG1 expression along with increased amyloid pathology (Hu et al., 2006; Kataria et al., 2019). BACE1 signaling impact on remyelination may be mediated through the neuregulin family, as selective deletion of BACE1 leads to subsequent NRG1 loss in peripheral injury (Hu et al., 2015; Nguyen et al., 2019). Although NRG1 can be cleaved by ADAMs, BACE1-specific cleavage appears necessary for NRG1 to signal myelin production. Yet, bypassing this pathway is possible, as promoting downstream protein kinase B (Akt) expression in oligodendrocytes rescues NRG1-associated myelin production in a BACE1-deficient model (Hu et al., 2013). Remyelination does not occur even in the presence of BACE1 in an APP-knockout (KO) model (Truong et al., 2019). On the other hand, in central nervous system the loss of Bace1 mainly disrupts neuroblast migration and maturation, resulting in an accumulation of neuroblasts and potential impairment of neuronal connectivity in the hippocampus (Benoit et al., 2023). γ-Secretase, another cleavage enzyme, is responsible for Aβ cleavage, and its inhibition decreases Aβ levels (Kounnas et al., 2010) subsequently promoting remyelination (Dovey et al., 2001). Inhibition of γ-secretase is associated with quicker disease recovery and milder pathology in a demyelinating animal model (experimental autoimmune encephalomyelitis, EAE), similar to multiple sclerosis (MS). Specifically, impairment if Notch1 signaling through γ-secretase inhibition promotes a pro-myelinating environment (Jurynczyk et al., 2008).

Collectively, amyloid-beta (Aβ) and upstream amyloid precursor protein (APP) exhibit both pro- and anti-remyelinating properties in a context dependent manner. The involvement of sAPPα, BACE1, and γ-secretase in regulating Aβ oligomer deposition in AD suggests a more central role in myelin regulation than previously described.

### 4.2 ApoE and lipid metabolism

Apolipoprotein E (ApoE) plays a significant role in various neurodegenerative conditions affecting myelin (Strittmatter and Roses, 1996). The APOE genotype, especially the ε4 allele, is associated with multiple sclerosis (MS) and altered white matter integrity (Rafiei et al., 2012). APOE ε2 is linked to impaired remyelination in MS but is protective against Alzheimer's disease (AD) and associates with higher myelin content (Corder et al., 1994; Carlin et al., 2000; Suri et al., 2013). APOE-deficient experimental models exhibit impaired remyelination and show altered disease progression attributed to cholesterol accumulation (Karussis et al., 2003; Cantuti-Castelvetri et al., 2018). ApoE interacts with triggering receptor expressed on myeloid cells 2 (TREM2) that is crucial for myelin repair by modulating lipid droplet formation (Takahashi et al., 2007; Petković et al., 2016; Wolfe et al., 2018). TREM2 is pivotal in phagocytosing myelin and cellular debris, particularly through the white matter-associated microglial phenotype (WAM) (Gouna et al., 2021). WAM, dependent on TREM2, shares genetic characteristics with disease-associated microglia (DAM) seen in transgenic AD mice (Keren-Shaul et al., 2017). WAM is APOE-independent in wild-type aging mice. However, in AD mouse models both TREM2 and ApoE are required for WAM development (Safaiyan et al., 2021). ApoE regulates lipoprotein lipase (LPL), which is crucial for microglial reparative functions, lipid uptake, and myelin-related lipid phagocytosis (Bruce et al., 2018; Pedrini et al., 2021). LPL deficiency is implicated in AD progression, and its administration elevates cellular Arg1 levels, linked to myelin repair (Ma et al., 2021). Colony-stimulating factor 1 receptor inhibition may enhance phagocytic capacity for remyelination (Wies Mancini et al., 2019). Upregulation of lipid receptors such as liver X receptors (LXR) promotes remyelination, reduces inflammation, and alleviates cholesterol overload (Nelissen et al., 2012). Furthermore, retinoid X receptor (RXR) signaling enhances ABCA1 and APOE expression, facilitating oligodendrocyte precursor cell and oligodendrocyte maturation, thereby improving cognitive function in AD (Cantuti-Castelvetri et al., 2018; Santos-Gil et al., 2021).

APOE has been shown to play a critical role in amyloid-beta (Aβ) deposition and plaque formation. APOE deletion in mouse models of amyloidosis leads to a significant reduction or failure in the deposition of Aβ plaques. This highlights APOE's crucial role in facilitating the accumulation of Aβ, a hallmark of AD pathology (Bales et al., 1999). Contrarily, another research revealed that conditional knockout (KO) of APOE specifically in microglial cells of 5xFAD mice results in an increase in plaque size without affecting total plaque numbers. This suggests a nuanced role of APOE in influencing plaque morphology through microglial-mediated mechanisms, potentially involving Aβ clearance or aggregation dynamics (Shi et al., 2020). Together, these studies underscore the complex involvement of APOE in AD pathogenesis, influencing Aβ aggregation and plaque characteristics through multiple cellular pathways, thereby implicating APOE as a potential therapeutic target for modifying disease progression.

ApoE mimetics have been found efficient in promoting myelin repair and inhibiting macrophage activity in the peripheral nervous system (Li et al., 2010). Modulation of ApoE-related signaling pathways, including synthetic agonists for liver X receptors (LXR) and retinoid X receptors (RXR), holds promise as remyelination promoting therapies as well (Santos-Gil et al., 2021). The FDA-approved RXR agonist, bexarotene, is linked to remyelination in AD mouse models and cognitive recovery in stroke-induced demyelination (Song et al., 2022). Furthermore, TREM2 potentially enters the brain from peripheral sites and directly influences the activity of oligodendrocyte precursor cells (OPCs) and oligodendrocytes (OLs) (Raha et al., 2016). Overall, regulation of ApoE-related signaling pathways emerges as a strategy to enhance cognition and promote remyelination in AD and other models of demyelination and vascular injury.

### 4.3 Tau and neurofilament proteins

Myelin impairment is an early event in tauopathies, followed by cognitive deficits. Remyelination has been demonstrated to mitigate cognitive decline in rTg4510 tauP301L mice (Jackson et al., 2018). In Alzheimer's disease (AD), tau becomes hyperphosphorylated and forms neurofibrillary tangles, influencing oligodendrocyte precursor cell (OPC) differentiation (Fressinaud et al., 2012; Ossola et al., 2016). Tau also binds to the cytoskeleton of oligodendrocytes (OLs) through the truncating tyrosine kinase Fyn, involved in tau phosphorylation (Lee J.-T. et al., 2004; Belkadi and LoPresti, 2008). Reduction of tau phosphorylation (p-tau), without altering total tau levels, enhances myelin repair and improves functional outcomes (Fu et al., 2020). Furthermore, tau phosphorylation at specific sites, such as Thr205 (pT205) and Ser202/Thr205 (AT8 epitope), is essential for microtubule remodeling during early neuronal development. This phosphorylation modulates tau's binding affinity to microtubules, facilitating necessary plasticity for axonal growth and dendritic branching, which are critical for cytoskeletal dynamics during initial cell growth phases (Hefti et al., 2019; Morris and Brady, 2022; Johnson and Stoothoff, 2004). Tau is associated with axonal neurofilament proteins (NFPs), where specific fractions like NFP2 and NFP5 play roles in OL lineage and development. The NFP to tubulin ratio may impact OL lineage, with NFP2 linked to OPC proliferation and NFP5, influencing OL maturation and differentiation (Fressinaud et al., 2012). Soluble Aβ oligomers can stimulate OL differentiation and induce myelin basic protein (MBP) expression through the Fyn/Ca2/CAMKII signaling cascade, implicating Fyn as a potential target for simultaneously modulating myelin regeneration and tau hyperphosphorylation (Quintela-López et al., 2019).

**Figure 1:**
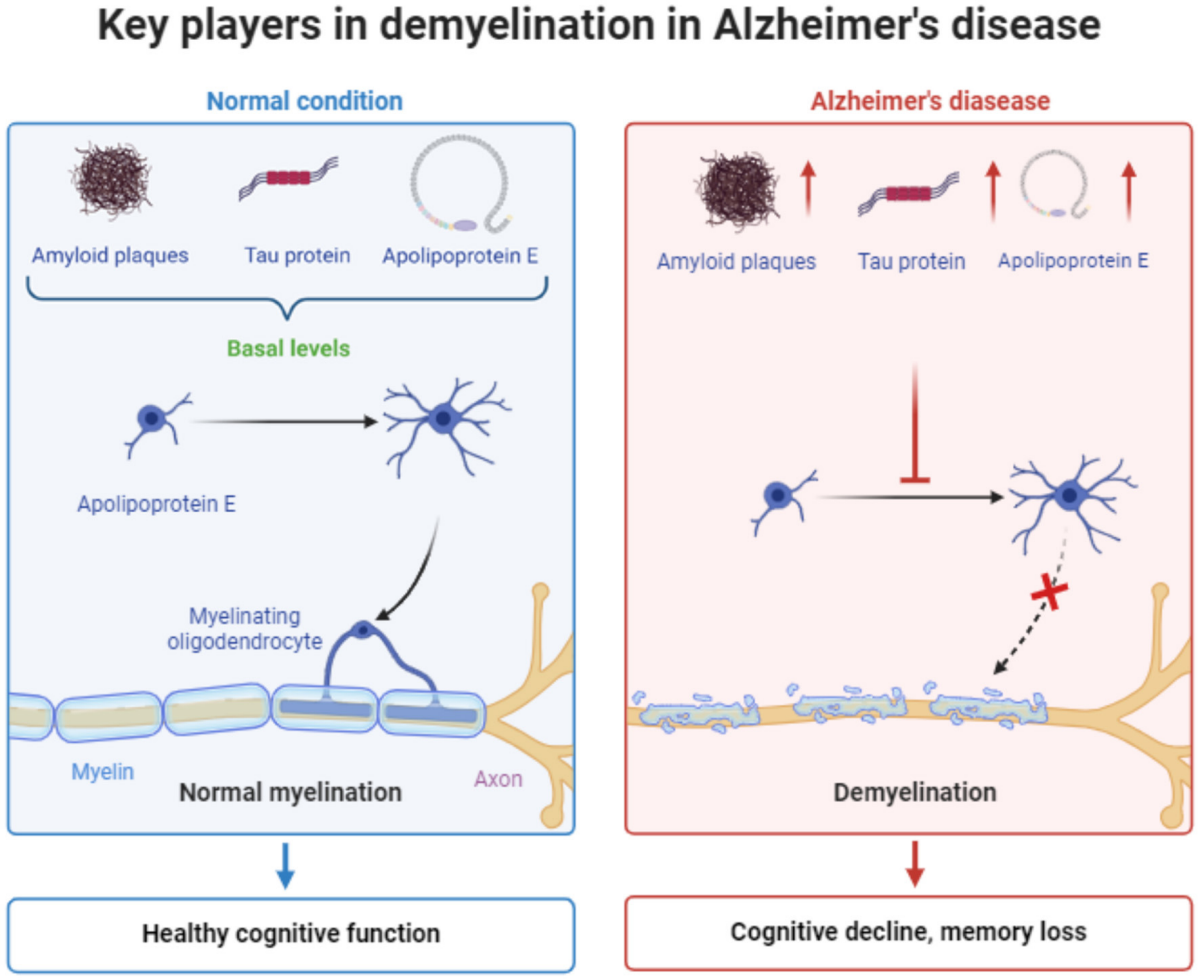
Schematic representation the main factors contributing to demyelination in Alzheimer's disease. The figure was created using the BioRender application.

Conclusively, significant endogenous factors like Amyloid-β/APP, tau and ApoE proteins consist key regulatory factors for myelin homeostasis, depending on their expression levels and structural properties. Indeed, basal levels of these factors, meaning Aβ concentration at picomolar to nanomolar range (Kamenetz et al., 2003; Deshpande et al., 2009; Yankner and Lu, 2009), non-hyperphosphorylated and low nanomolar concentration of tau (Avila et al., 2004; Iqbal et al., 2005; Goedert and Spillantini, 2006) and low ApoE4/ApoE3 ratio (Huang and Mahley, 2014; Mahley and Huang, 2012; Kim et al., 2014) are positive regulators of myelination, while their increased expression or disrupted structure during Alzheimer's Disease progression lead to myelin destabilization and neuronal deficits (Figure 1).

### 4.4 PI3K signaling

The PI3k signaling cascade, along with its associated components, is implicated in promoting remyelination and intersects with pathways involved in Alzheimer's disease (AD) pathology. Akt, a downstream effector of PI3k, has been associated with BACE1 cleavage, suggesting that Akt expression could potentially enable BACE1 inhibitors to suppress amyloid without affecting remyelination (Hu et al., 2013). Activation of the PI3k pathway, either directly or indirectly, has been shown to enhance remyelination (Hochmeister et al., 2012; Hu et al., 2013; Liu et al., 2017; Kopec et al., 2019). For example, the Chinese herbal compound Shen-zhi-ling (SZL) oral liquid and the FDA-approved AD treatment donepezil increase PI3k and Akt mRNA expression, along with enhancing mTOR-positive cells and myelin-related proteins in AD mouse models (Qin et al., 2021). Donepezil promotes oligodendrocyte precursor cell (OPC) differentiation to oligodendrocytes (OLs), myelin sheath formation, and the upregulation of myelin-specific proteins, whereas rivastigmine, another AD therapeutic agent, shows less impact on OPCs and myelination compared to donepezil (Imamura et al., 2017; Cui et al., 2019). The exact remyelination mechanism of donepezil is not fully understood, but modulation of the PI3k/Akt/mTOR pathway is believed to contribute (Imamura et al., 2017). Furthermore, low doses of a PI3k inhibitor can upregulate OPCs and encourage OL maturation. However, whether this effect is PI3k-specific or results from off-target effects on Wnt and RAF-MAPK signaling remains uncertain. Overall, modulating the PI3k/Akt pathway may be beneficial for promoting remyelination, particularly in the context of AD. Further investigation of PI3k and Akt in both myelination and AD contexts could provide valuable insights into relevant biological pathways contributing to AD (Rivera et al., 2022). Another study showed that continued activation of extracellular-regulated kinases 1 and 2 (ERK1/2) in cells of the oligodendrocyte lineage leads to an expedited repair of myelin after injury. This sustained activation alone is adequate for producing thick myelin sheaths around remyelinated axons in the adult mouse spinal cord. These findings propose an interesting model in which ERK1/2 MAP kinase signaling serves as a regulator for myelin thickness, guiding oligodendrocytes to generate appropriate amounts of myelin for the axons (Fyffe-Maricich et al., 2013).

### 4.5 Iron levels

Maintaining proper iron balance is crucial for glial function and myelination, and recent literature suggests a connection between dysfunctional iron homeostasis and Alzheimer's disease (Gleason and Bush, 2021; Peng et al., 2021; Tran et al., 2022). Neuroimaging studies demonstrated associations between iron accumulation and myelination in aging, whereas iron levels were associated with tau accumulation in AD patients (Möller et al., 2019; Spotorno et al., 2020; Khattar et al., 2021). Cerebrospinal fluid levels of iron transport proteins also correlate with cognitive decline in AD (Tran et al., 2022). Meanwhile, there are conflicting evidences regarding the impact of the activation of iron storage and proteins transportation in AD progress. Ferritin, an iron storage protein, supports remyelination and oligodendrocyte (OL) function through microglial H-ferritin release (Schonberg et al., 2012; Zhang et al., 2021). However, excess ferritin in inflammatory environments may be toxic to OLs (Zhang et al., 2021). Excess iron is linked to increased amyloid precursor protein (APP) in animal models, affecting cleavage processes, but APP, in turn, stabilizes the iron export protein ferroportin (Fpn), crucial for OL maturation and myelination (Schulz et al., 2012; Wong B. X. et al., 2014; Tran et al., 2022). Divalent metal transporter 1, an iron import protein, is involved in APP processing and is associated with reduced oligodendrocyte precursor cell (OPC) maturation when deleted (Zheng et al., 2009; Cheli et al., 2018). Additionally, transferrin, another iron importer, binds to tau and is associated with phosphorylated tau in AD patients, while enhancing microglial phagocytic capacity and supporting OL maturation (Jahshan et al., 2016; Hoshi et al., 2021). The complex interplay of iron-related proteins and their effects on myelination and AD underscores the need for further research in this field (Adamo et al., 2006; Carden et al., 2019).

### 4.6 Neuregulin

Neuregulin, particularly neuregulin-1 (NRG1), is essential for oligodendrocyte development and the regulation of myelination, processes that are notably disrupted in Alzheimer's disease. NRG1 is crucial for the maintenance and survival of oligodendrocytes, which are responsible for the formation of myelin sheaths around axons. In AD, the dysregulation of NRG1 leads to significant myelin abnormalities, contributing to cognitive decline and neuronal dysfunction (Ledonne et al., 2018). The disruption in neuregulin-ErbB signaling pathways in AD models exacerbates myelin damage. This imbalance in signaling pathways results in impaired oligodendrocyte function and reduced myelin production, thereby accelerating neuronal degeneration and AD pathology (Ou et al., 2021). Additionally, reduced levels of neuregulin in AD brains correlate with decreased myelin sheath thickness and oligodendrocyte loss. This loss of oligodendrocytes is a critical factor in the progression of AD, as these cells are pivotal for myelin maintenance and repair (Chen et al., 2013). Research into the molecular mechanisms by which neuregulin regulates myelination through its interactions with ErbB receptors on oligodendrocytes showed that perturbations in neuregulin-ErbB signaling lead to the myelin defects observed in AD, including the thinning of myelin sheaths and reduced myelin integrity, which are hallmarks of the disease (Xie and Zheng, 2013). Collectively, these studies highlight the critical role of neuregulin in the regulation of myelination and its significant contribution to the myelin abnormalities seen in Alzheimer's disease.

In summary, there are numerous target pathways that intersect with both myelin repair and Alzheimer's disease pathophysiology, including APP processing, ApoE signaling, and tau-Fyn processing. These pathways can be recruited to promote myelin repair targeting three key procedures: (1) oligodendrocyte precursor cell (OPC) proliferation, (2) oligodendrocyte (OL) maturation, and (3) myelin sheath production. These targets could potentially be utilized not only for myelin repair but also to prevent initial myelin damage. However, further research is needed to ascertain which aspects of these processes are most affected in AD, whether myelin repair dysfunction occurs earlier in the disease course than previously thought, and which of the identified pathways can effectively address myelin pathology and potentially restore myelin function.

## 5 The therapeutic potential of neurotrophins in AD

The therapeutic benefit of NGF and BDNF has been tested in many neurodegenerative diseases treatment, including Alzheimer's disease. However, the use of recombinant proteins has faced challenges due to their brief lifespan in the bloodstream and their poor capacity to penetrate the Blood Brain Barrier (BBB). A clinical trial administering NGF directly into the basal forebrain of ten Alzheimer's patients demonstrated promising indications of enhanced cognitive and neuronal function (Eyjolfsdottir et al., 2016). An ongoing clinical trial is exploring gene therapy with AAV2-BDNF for early Alzheimer's, building on positive outcomes from preclinical animal studies that suggest the neuroprotective effects of BDNF (Nagahara et al., 2013). The limited success of recombinant neurotrophin use has prompted the development of alternative tools such as small peptide mimetics, agonistic monoclonal antibodies, and small molecules activating the NGF/TrkA and BDNF/TrkB pathways (Nordvall et al., 2022).

Decline in BDNF levels in the hippocampus of Alzheimer's disease patients was strongly associated with disease progression. BDNF plays a crucial role in supporting the basal forebrain cholinergic system, which undergoes degeneration in AD. Reduced levels of BDNF in mRNA and protein level have been detected in the brains of AD patients, and studies have demonstrated that increasing BDNF levels can enhance memory and cognitive function in both AD patients and mouse models (Ibrahim et al., 2022). Moreover, the interaction between Amyloid β and protein kinase A (PKA) activation can diminish BDNF expression, resulting in reduced synaptic plasticity and cognitive function. Additionally, BDNF stimulation has been observed to induce the de-phosphorylation of tau, another protein implicated in AD pathology, and to redirect amyloid precursor protein (APP) processing toward a non-amyloidogenic pathway (Jiao et al., 2016; Nigam et al., 2017). These findings suggest that enhancing BDNF signaling may represent a promising therapeutic strategy for AD (Azman and Zakaria, 2022; Gao et al., 2022). Furthermore, BDNF-TrkB signaling has been shown to regulate adult hippocampal neurogenesis, a process severely affected in AD (Vilar and Mira, 2016; Colucci-D'Amato et al., 2020; Salta et al., 2023). Thus, novel small molecules capable of activating TrkB and its downstream signaling pathways could hold therapeutic potential for AD.

Many studies have focused on the development of neurotrophin mimetics. The TrkB agonist/stimulator LM22A-4 was identified by the use of structural information obtained from BDNF in an *in silico* screening process. The compound demonstrated efficacy in preventing neuronal degeneration *in vitro* comparable to BDNF (Massa et al., 2010). Another *in silico* screening revealed LM22B-10, which binds to both TrkB and TrkC receptors. In aged mice, LM22B-10 activated hippocampal and striatal TrkB and TrkC receptors, along with their downstream signaling pathways, leading to increased dendritic spine density in the hippocampus (Yang et al., 2016). To enhance bioavailability, structural modifications led to the development of PTXBD10-2. This compound was found to activate TrkB and TrkC receptors and enhance cholinergic neurite integrity in the basal forebrain in a mouse model of Alzheimer's disease, as evidenced by increased choline acetyltransferase (CHAT) presence (Gonzalez et al., 2022). An additional compound, the dimeric dipeptide GSB-106, was designed from a loop region of BDNF and has demonstrated neuroprotective and antidepressant effects (Vakhitova et al., 2021). Moreover, LM11A-31, a p75NTR pan-neurotrophin receptor modulator, has shown beneficial effects in a mouse model of the disease during the mid to late stages. A modified formulation is currently undergoing clinical trials (Simmons et al., 2014) (Clinical Trial Number: NCT03069014).

Natural products have been a valuable source of compounds, which have been found to regulate Trk signaling. Examples include gambogic amide, asiaticoside, and sarcodonin G, which activate TrkA, while deoxygedunin acts on TrkB (Jang et al., 2007, 2010; Cao et al., 2018; Nalinratana et al., 2019). Despite their diverse chemical potential, natural products pose challenges in understanding structure-activity relationships. One well-studied compound is 7,8-dihydroxy flavone (7,8-DHF), a TrkB agonist, with derivatives like the benzimidazole derivative (CF3CN) showing improved pharmacokinetic properties. 7,8-DHF mimics BDNF, offering cognitive and antidepressant benefits, neuroprotection, neuroplasticity, and neurotrophic properties (Jang et al., 2010; Zeng et al., 2012; English et al., 2013; Zhang et al., 2015; Chen et al., 2021). In summary, there are many options to pharmacologically target neurotrophin signaling. Molecular analyses and *in vivo* assessment will unravel their potential benefit in oligodendrocytes to promote (re-)myelination in a diseased context.

## 6 The role of neurotrophins in oligodendrocyte dynamics

Neurotrophins have a significant impact on cells of the oligodendrocyte lineage in the central nervous system (CNS). More specifically, Brain-Derived Neurotrophic Factor (BDNF) and Neurotrophin-3 (NT-3), are known to promote the survival and differentiation of oligodendrocyte precursor cells (OPCs) into mature oligodendrocytes (Rubio et al., 2004; Pukos et al., 2018; Siebert and Osterhout, 2021). BDNF and NT-3, through their respective receptors (TrkB and TrkC), have been implicated in promoting myelination in the CNS. Neurotrophins are involved in the bidirectional communication between neurons and oligodendrocytes (Berghuis et al., 2004; Jang et al., 2019). Neurons release neurotrophins that, in turn, affect the maturation and myelination of oligodendrocytes (Wan et al., 2010; Xiao et al., 2011). The interaction between axons and oligodendrocytes mediated by neurotrophins is crucial for the proper development and maintenance of myelinated fibers. Neurotrophins show neuroprotective effects on oligodendrocytes, safeguarding them from injury or degeneration (Pukos et al., 2018). For example, BDNF has been shown to protect oligodendrocytes from glutamate-induced excitotoxicity (Almeida et al., 2005). Neurotrophins activate various signaling pathways, including the PI3K/Akt and MAPK/Erk pathways, which are implicated in oligodendrocyte survival, proliferation, and differentiation (Huang and Reichardt, 2001). These signaling cascades contribute to the overall impact of neurotrophins on oligodendrocyte biology. Collectively, neurotrophins play a multifaceted role in regulating oligodendrocyte populations by influencing their survival, differentiation, myelination, and interactions with axons. Understanding these mechanisms is crucial for elucidating the complex interplay between neurons and glial cells in the CNS and may have implications for therapeutic strategies aimed at promoting myelin repair and regeneration in pathological conditions involving demyelination.

## 7 BDNF is a key player in oligodendrocyte proliferation, differentiation and maturation

**Figure 2:**
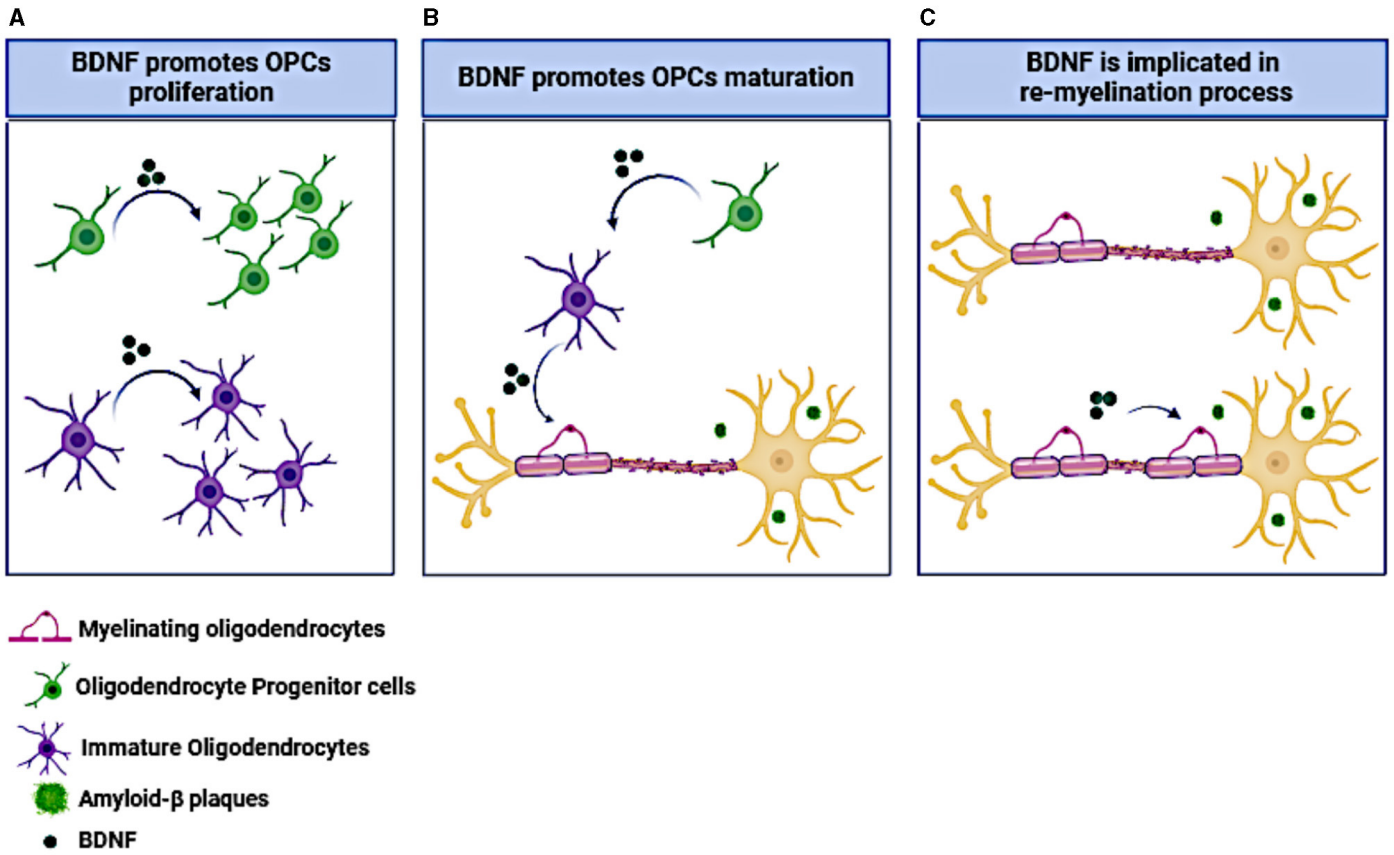
The effect of BDNF on OPCs and oligodendrocytes. **(A)** BDNF administration promotes OPCs and immature oligodendrocytes proliferation. **(B)** BDNF induces OPCs maturation toward myelinating oligodendrocytes. **(C)** BDNF administration facilitates neuronal re-myelination under neurodegenerative conditions like the presence of amyloid-β plaques. The figure was created using the BioRender application.

Beside the neuronal support, the BDNF/TrkB signaling is well documented to promote the OPC proliferation, differentiation and myelination (Djalali et al., 2005; VonDran et al., 2011; Tsiperson et al., 2015), reviewed by Fletcher et al. (2018) and Schirò et al. (2022). Phosphorylation of the neurotrophin receptor TrkB, expressed on the cell membrane of oligodendrocytes, appears to be a crucial factor in the myelination process of the central nervous system (Xiao et al., 2012; Wong et al., 2013). BDNF acts through this receptor and enhances myelination in the CNS and increases the density of OPCs both *in vitro* and *in vivo* (McTigue et al., 1998; Du et al., 2003; Fulmer et al., 2014; Wong A. W. et al., 2014). The effects of BDNF on oligodendrocytes and myelination are represented in Figure 2.

Oligodendrocytes express both the TrkB and p75NTR receptors, indicating their responsiveness to both mature brain-derived neurotrophic factor (BDNF) and pro-BDNF (Tsiperson et al., 2015). Pro-BDNF, appears to exert inhibitory effects on oligodendrocytes. Specifically, it reduces cell proliferation and migration in a line of OLN-93 oligodendrocytes through p75NTR signaling (Liu et al., 2018). Interestingly, the expression of p75NTR does not seem to be necessary to induce myelination. Only BDNF, not pro-BDNF, promotes myelination and proliferation in oligodendrocyte cultures (Du et al., 2006). BDNF-deficient mice show reduced levels of various myelin proteins, including MAG, MBP, and PLP, along with a deficit in the production of the oligodendrocyte lineage. Moreover, the loss of BDNF appears to specifically impact oligodendrocytes, without affecting astrocytes or microglia (VonDran et al., 2011). Notably, the effect on myelination seems to be directly mediated by the TrkB receptor, while the proliferation of OPCs is independently influenced by TrkB, as it can be also stimulated by the expression of TrkC (Wong et al., 2013).

It is also of paramount importance to note that the use of BDNF to promote OPCs proliferation and differentiation should be done post-peak myelination to ensure that developmental mechanisms of oligodendrocyte-lineage cells are preserved (Fancy and Chan, 2011; Xiao and Wong, 2017). During early development, there are specific patterns of BDNF signaling that regulate the proliferation and differentiation of OPCs. Premature or excessive BDNF signaling could disrupt these developmental processes, potentially leading to abnormalities in myelination patterns (Baydyuk and Xu, 2014; McTigue and Tripathi, 2008). If BDNF signaling persists at high levels beyond the critical developmental period, it might interfere with this pruning process, leading to a less efficient or overly dense myelinated network and hypermyelination. This excessive formation of myelin sheaths could potentially disrupt the balance and function of neuronal circuits (Nave and Werner, 2014).

BDNF release has been suggested to act as a signaling mechanism for adaptive myelination. In various *in vitro* studies, while BDNF signaling may influence the proliferation and differentiation of OPCs, as well as to promote developmental myelination (Fletcher et al., 2018). Additionally, BDNF sensitizes the myelination process of NMDA receptor modulation in neuron-oligodendroglia co-cultures (Lundgaard et al., 2013). Oligodendrocytes lacking the BDNF receptor TrkB in mice display a developmental delay in myelin thickness, indicating a direct yet temporary *in vivo* impact of BDNF on oligodendrocytes (Wong et al., 2013). These mice, with ablated TrkB in a sub-population of OPCs, show increased OPC numbers attributed to higher proliferation (Wong et al., 2013). BDNF is also implicated in adaptive myelination resulting from neuronal stimulation, with effects partially mirroring developmental outcomes. Studies involving inducible conditional knockout mice reveal that inhibiting TrkB in OPCs or activity-regulated BDNF in stimulated neurons prevents the increase in OPC proliferation, oligodendrocyte generation, and myelin thickness (Geraghty et al., 2019). These findings suggest that BDNF may act as a cue transmitting neuronal activation signals to OPCs and/or oligodendrocytes. It is crucial to confirm the conservation of BDNF's role in AD models, its applicability across different brain regions (Du et al., 2003), and its interactions with other adaptive myelination cues such as glutamate *in vivo* (Lundgaard et al., 2013).

BDNF also enhances neurogenesis and neuronal wellbeing primarily through its interaction with the TrkB receptor, activating pathways such as MAPK/ERK, PI3K/Akt, and PLC-γ. These pathways promote the proliferation, differentiation, and survival of neural progenitor cells, as well as synaptic plasticity (Li et al., 2008; Huang and Reichardt, 2001). BDNF also protects neurons from stress, supports metabolic functions, and fosters axonal and dendritic growth (Dieni et al., 2012; Liu et al., 2014). Improved myelination observed with increased BDNF levels is probably an effect of heightened neuronal demand for myelin, driven by the growth and maturation of neurons, rather than direct effects on oligodendroglial-lineage cells (Mitew et al., 2010; Fields, 2015). Enhanced neuronal activity and connectivity further stimulate myelination, creating a supportive environment for oligodendrocyte function (Boulanger and Messier, 2014).

Despite its promising re-myelinating activity, BDNF fails in clinical trials likely due to its inability to cross the blood-brain barrier, its short half-life and low pharmacokinetic profile (Poduslo and Curran, 1996). Several studies have tried to develop synthetic agonists for specific NT receptors. Examples include BDNF-mimics that target specifically TrkB receptor and not p75NTR like tricyclic dimeric peptide 6 (TDP6), a small multicyclic peptide that structurally mimics a region of BDNF that binds TrkB (Wong A. W. et al., 2014) and LM22A-4, a non-peptide small molecule that acts as a TrkB agonist to promote remyelination in cuprizone mouse model (Nguyen et al., 2019).

## 8 BDNF-mediated oligodendrogenesis and re-myelination: a promising therapeutic approach for Alzheimer's disease

Although BDNF was shown critical for neurogenesis and neuronal survival in AD models (reviewed by Numakawa and Kajihara, 2023a,b), little is known about its benefits in myelination in AD. Noteworthy, BDNF exhibits a strong pro-myelination effect in the cuprizone model of demyelination indicating its potential use as a therapeutic molecule against myelin diseases like Multiple Sclerosis (Nguyen et al., 2019). As described above myelin deficits exacerbate neurodegeneration and increase the risk for the onset of AD. As a result, the administration of promyelination compounds could serve as a preventive strategy against neurodegeneration. Evidence indicates that TrkB agonists exhibit a pro-differentiation and pro-myelinating impact in 8–10 week-old *CNPase*Cre+/– × *TrkB*fl/fl mice [a mouse model in which TrkB receptor has been deleted only in CNPase-expressing cells using Cre-loxP system (Lappe-Siefke et al., 2003)] and cuprizone mouse model (Fletcher et al., 2018; Nguyen et al., 2019). CNPase is a protein expressed predominantly in oligodendrocytes, where it plays a critical role in myelin formation and maintenance, regulation of microtubules, intracellular signaling, cytoskeletal organization, and energy metabolism (Verrier et al., 2013). The CNPase promoter is selected for conditional knock-out of TrkB receptor gene in oligodendrocytes, in order to study the receptor's functions in myelinating cells, in a cell-specific manner. CNPase expression begins at low levels during early postnatal development, progressively increases during the myelination phase, and then stabilizes at high levels in adulthood, making it an ideal promoter for studying gene function during both development and in the mature central nervous system (Rao and Dawson, 2000). These findings support a potential therapeutic use of BDNF in combating demyelination within the context of AD. Until recently there was no systemic analysis of the expression profile of neurotrophin receptor in oligodendrocytes under AD conditions, information valuable for the development of new therapeutic strategies against de-myelination in this disease. Recent study of our lab focused on characterization of neurotrophin receptors in OPCs. Our findings show that OPCs resided in the hippocampus of 5xFAD mice express TrkB, TrkC, and p75 receptors but not the TrkA (Zota et al., 2024). Considering the observed neuroprotective and pro-myelinating effects of the TrkB agonist LM22A-4 in models of demyelination and traumatic brain injury (Fletcher et al., 2021), a promising hypothesis is that targeting TrkB with novel agonists could serve as a drug-based strategy to enhance myelin, complementing amyloid and tau-centered therapies in AD.

Currently, the licensed drugs for AD target acetylcholinestase, providing limited relief of symptoms. It is therefore crucial to find “disease-modifying” treatments (Zhu et al., 2013; Birks and Harvey, 2018; Secnik et al., 2020). The first FDA-approved drug for AD targets the Aβ peptide, but it has sparked controversy among experts (Howard and Liu, 2020; Schneider, 2020; Knopman et al., 2021). Many other attempts to target Aβ plaques or peptides have either failed or are in late stage clinical trials (Cummings et al., 2019, 2020, 2021; Liu et al., 2019). Delivery of BDNF through intracerebroventricular injections or other non-invasive methods has also been unsuccessful due to side effects or low efficiency (Givalois et al., 2004; Kopec et al., 2019). The evidence discussed in this study suggests that a small, blood brain barrier-permeable molecule that acts as a BDNF mimetic, could counteract the harmful effects of Aβ and concomitantly induce re-myelination in AD.

Over the past decade, our research group has elucidated the molecular mechanism through which the endogenous neurosteroid dehydroepiandrosterone (DHEA), produced within the brain, shields neurons from apoptosis (Charalampopoulos et al., 2004). Remarkably, DHEA was found to bind and activate all Trk and p75NTR neurotrophin receptors across different neuronal cell types (Lazaridis et al., 2011). These findings led us to hypothesize that DHEA might have acted as an ancestral neurotrophic factor, promoting neuronal survival in ancient, less complex nervous systems (Pediaditakis et al., 2015). However, the potential long-term clinical utility of DHEA as a neuroprotective therapeutic is limited due to its numerous secondary effects stemming from its binding to various steroid and neurotransmitter receptors, as well as its central role as a precursor steroid in the synthesis of androgens and estrogens (Charalampopoulos et al., 2008).

**Figure 3:**
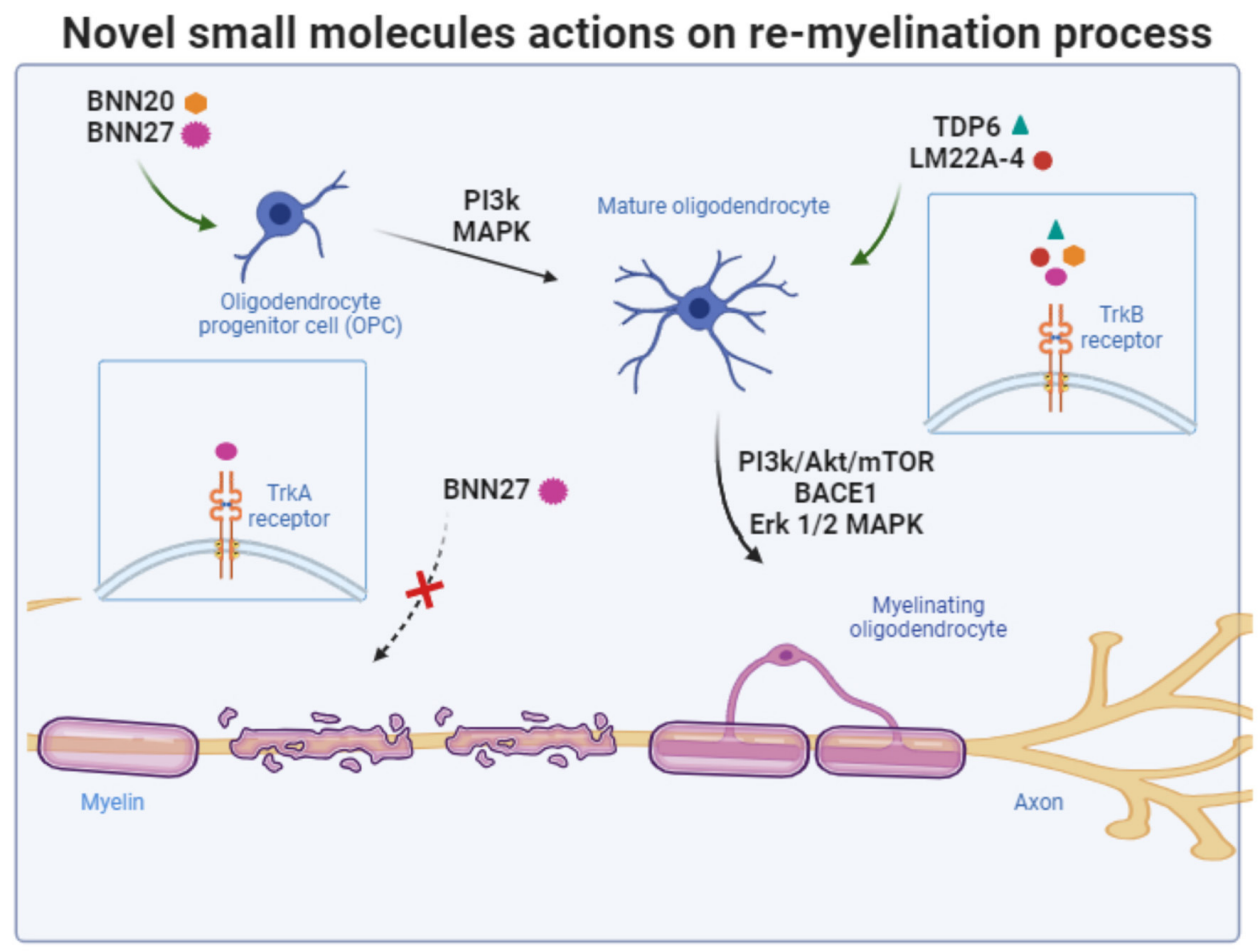
Schematic summary of the targets of novel small molecules and the signaling pathways that are recruited to protect oligodendrocytes and promote re-myelination process. The figure was created using the BioRender application.

Synthetic versions of DHEA, devoid of endocrine effects, form a novel group of compounds capable of crossing the blood-brain barrier and binding to neurotrophin receptors, providing neuroprotective effects. Researchers have developed 17-helix epoxy analogs of DHEA, such as BNN20 and BNN27, with modifications at positions C3 and C17. These analogs, termed “steroidal microneurotrophins,” function as activators of neurotrophin receptors and can potentially have therapeutic applications in neurodegenerative diseases. Among them, BNN20 that binds to TrkA and TrkB receptors, an epoxidation-modified analog, has demonstrated neuroprotective effects *in vitro* and *in vivo*. BNN20 protects dopaminergic neurons, reduces inflammation, and promotes oligodendrocyte differentiation in mouse models of Parkinson's disease and demyelination induced by LPS (Calogeropoulou et al., 2009; Panagiotakopoulou et al., 2020; Kalafatakis et al., 2021). Research also suggests that BNN27 that activates TrkA and p75 receptors enhance memory in AD models, interacts with the cholinergic system, protect oligodendrocytes and myelin in demyelinating disorders, and provide therapeutic benefits for diabetic retinopathy by addressing neurodegeneration and inflammation (Bonetto et al., 2017; Pitsikas and Gravanis, 2017; Ibán-Arias et al., 2018). To date, comprehensive investigations into novel small molecules specifically targeting TrkB receptor, capable of selectively mimicking the functions of BDNF, are lacking. Such molecules hold promise as potential therapeutic agents with the capacity to exhibit both remyelinating and neuroregenerative properties (Figure 3). Recent efforts to design and synthesize such BDNF mimetics are under way. These novel BDNF-mimetics activate selectively TrkB receptor and its downstream pathways such as PI3k and MAPK signaling cascades paving the way for their potential use to promote re-myelination process (Antonijevic et al., 2024; Narducci et al., 2023; Charou et al., 2024).

## 9 Conclusion

In conclusion, the mechanisms underlying myelin breakdown in Alzheimer's disease and the potential therapeutic benefits of enhancing myelin renewal are areas of ongoing investigation (Lee et al., 2012; Mei et al., 2016; Depp et al., 2023). The complex involvement of neurotrophins in promoting oligodendrocyte precursor cell (OPC) proliferation and remyelination, in addition to their neurogenic and neuroprotective effect, offers a promising avenue for alternative therapeutic approach in Alzheimer's disease (AD). Specifically, based on the aforementioned data we suggest that selectively inducing BDNF signaling to enhance oligodendrogenesis and myelination could hold promise as a therapeutic approach against amyloid-β-induced toxicity in AD. The development of novel BDNF mimetics paves the way for a drug-based strategy to enhance myelination, complementing existing αβ- and tau-centered therapies for AD. Further research into the efficacy and safety of such interventions is imperative to potentially address the complex pathophysiology of AD and improve treatment outcomes for affected individuals.
